# Influence of Some Microchanges Generated by Different Processing Methods on Selected Tribological Characteristics

**DOI:** 10.3390/mi13010029

**Published:** 2021-12-26

**Authors:** Gheorghe Nagîț, Laurențiu Slătineanu, Oana Dodun, Andrei Marius Mihalache, Marius Ionuț Rîpanu, Adelina Hriţuc

**Affiliations:** Department of Machine Manufacturing Technology, Gheorghe Asachi Technical University of Iasi, 700050 Iasi, Romania; nagit@tcm.tuiasi.ro (G.N.); slati@tcm.tuiasi.ro (L.S.); oanad@tcm.tuiasi.ro (O.D.); andrei.mihalache@tuiasi.ro (A.M.M.); marius.ripanu@tuiasi.ro (M.I.R.)

**Keywords:** machining methods, surface layer, microchanges, wear, friction coefficient, erosion, measurement, empirical mathematical model

## Abstract

Different processing methods can change the physical–mechanical properties and the microgeometry of the surfaces made by such processes. In turn, such microchanges may affect the tribological characteristics of the surface layer. The purpose of this research was to study the tribological behavior of a test piece surfaces analyzing the changes on the values of the coefficient of friction and loss of mass that appear in time. The surfaces subjected to experimental research were previously obtained by turning, grinding, ball burnishing, and vibroburnishing. The experimental research was performed using a device adaptable to a universal lathe. Mathematical processing of the experimental results led to the establishment of power-type function empirical models that highlight the intensity of the influence exerted by the pressure and duration of the test on the values of the output parameters. It was found that the best results were obtained in the case of applying ball vibroburnishing as the final process.

## 1. Introduction

It is known that during the first period of equipment use, the surface roughness decreases gradually to certain values that correspond to the normal use conditions.

Due to the reduction in the surface asperities heights, a change in the friction coefficient values is expected. On the other hand, the surface roughness reduction is accompanied by removal of the material from the two parts involved in relative movement to each other.

All these processes take place at the micro-space level, in a surface layer with a thickness of, at most, several hundred micrometers, as the parameters for evaluating the height of the roughness of the processed surfaces can reach values less than 1 µm.

Many experimental investigations aimed to highlight the influence exerted by the previously applied machining process on the tribological behavior of the surfaces thus obtained. Some of these investigations took into account the processes of ball burnishing and ball vibroburnishing as processes capable of affecting the tribological characteristics of the surfaces of the parts. Other researches were directed at defining the experimental conditions to be used when experimentally assessing the tribological behavior of the burnished or vibroburnished surfaces.

Thus, in the last decades of the previous millennium, in the former Soviet Union, I.G. Schneider and his collaborators developed ample researches concerning the vibroburnishing processes and the functional properties of surfaces obtained using such machining processes. In 1982, Schneider published a monograph in which the functional properties of the so-called surfaces with regular microrelief were analyzed using the methods accessible at that time [1].

Another researcher from the Soviet Union who investigated the vibroburnishing process was L.G. Odintsov. He and his collaborators published papers in which this subject was approached, and a monograph concerning the hardening and finishing surfaces created by plastic deformation processes in surface layers up to several hundred micrometers thick was elaborated [2].

Loh et al. (1990) studied the influence of the ball burnishing process conditions on surface hardening in the case of specimens made of AISI 1045 steel [3]. They noticed the possibility of increasing the hardness by 68% and obtaining the maximum hardness beneath the burnished surface, to depths with values less than 150 µm.

Kim et al. simulated the experimental conditions of a tribological test based on using a reciprocating tribometer and a specimen pneumatically pressed onto the disk counterface sample, corresponding to a block-on-ring type of test [4]. As wear process output parameters, the wear depth and the wear rate were taken into consideration. The research showed good agreement of the experimental results with the results obtained using the simulation, by means of finite element analysis, for the material pair of interest.

El-Tayeb et al. considered using a tribo-test machine. The load was ensured using a first-order lever [5] to investigate the tribological behavior of burnished cylindrical specimens made of aluminum Al-6061. They found a certain combination of values corresponded to the burnishing process input factors, which ensured that the best behavior of the tested material was obtained for the tested friction coefficient values. The experimental research was performed in dry contact and lubricated contact conditions, on specimens made of aluminum 6060 and stainless-steel counterface cups.

Wojciechowski and Nosal addressed the problem of scuffing resistance in the case of test sample surfaces previously obtained by cold burnishing processes [6]. They proposed that the surface layer energy accumulation should be considered. Their experimental tests showed that an increase in surface layer energy accumulation leads to a decrease in scuffing resistance. The surface layer considered in manufacturing engineering is usually less than 1 mm thick, and is frequently even less than 500 µm.

Low took the use of a tribo-test machine, in the presence of a first-order lever, into consideration [7]. He used a 316 stainless steel, 60 mm diameter cup as a counterface, while the specimen was pressed onto the counterface using weights placed at the ends of the lever.

Li and Han investigated how the recrystallization of the Ni_3_Al-based single crystal alloy IC6SX is affected when various surface mechanical treatments are applied [8]. Dry sandblasting, wet sandblasting, indentation, shot blasting, and burnishing were used as mechanical treatments. The research showed that the deformation amount was more pronounced when burnishing and dry sandblasting were applied than that generated by applying other superficial mechanical treatments. At the same time, an observation was made, according to which the recrystallization did not affect the mechanical properties of the investigated alloy.

Ovali and Akkurt developed experimental research to compare the results of using burnishing and other hole surface finishing processes in the case of specimens made of brass materials [9]. They noticed that the highest hardness and the best surface quality were obtained when applying the burnishing process.

Al-Saeedi et al. proposed using an adaptive neuro-fuzzy inference system to predict the workpiece hardness and roughness of the surfaces obtained using a roller burnishing process [10]. Their considerations were valid for the parts made of polyoxymethylene.

Lewandowski investigated the wear behavior of cast iron EN-GJSFP-500-7 after applying a burnishing process when using solid lubricant [11]. A tribological test based on the block-on-ring work schema was used. He noticed that better results for the tribological properties and surface roughness parameters were obtained when using machine oil.

Zaleski designed and used a tribological testing stand in which the charging force was applied to the end of a third-order lever using weights of known value [12]. He used such a stand to study the wear behavior of surfaces processed by grinding, and vibratory and shot peening, respectively.

An investigation of the wear of burnished specimens made of low-density polyethylene was conducted by Janczewski et al., using a ball-on-disc test [13]. They noticed improved behavior of the burnished surfaces as compared to the surfaces obtained by milling only. The experimental results proved that there was a decrease in the wear rate by 58%.

Krasnyy et al. investigated the wear and fretting resistance in the case of surfaces previously obtained by applying a vibroburnishing process and presenting certain regular microgroove patterns [14]. They aimed to improve the parts’ operation performances acting on the values of the parameters that characterize the groove patterns. In another paper, Krasnyy and Maksarov presented the results of research that aimed to highlight the tribotechnical characteristics of surfaces obtained by vibroburnishing [15]. The investigated surfaces presented regular microgeometric relief, and, in certain situations, they proved to have high wear resistance compared to the wear resistance of another finishing process.

Takada and Sasahara aimed to investigate the influence exerted by the shape of the frictional stir burnishing tool on the hardness of the surface layer, on the residual stress, and on the values of the surface roughness parameter *Ra* [16]. It was confirmed that a lower roughness value could be obtained when the tool diameter was larger. However, an increase in the thickness of the microhardened layer was observed for lower values of the tool tip radius.

Dzierwa and Markopoulos addressed the issue of the influence of the ball burnishing process on the topography and tribological properties of hardened steel [17]. Experimental research has confirmed the possibility of reducing the root mean square height of the surface *Sq* from 0.522 µm to 0.051 µm and increasing the wear resistance by using a ball burnishing process.

The integrity of the surface achieved when using micro-textured ball-end milling was studied by Yang et al. [18]. Empirical mathematical models were determined to highlight the influence exerted by different input factors of the ball-end milling process on the hardness of HV and the values of the roughness parameter *Ra* of the processed surfaces.

Hou and Li investigated the influence of micro-hardness changes on a titanium alloy’s friction and wear characteristics [19]. For the experimental research, they used the characteristics of the ball-on-disc contact test. According to one of the conclusions of their research, the effects of previous processing can be observed on a surface layer thickness of less than 100 µm, and these microchanges can significantly affect the wear and friction behavior of the specimen material.

The above information, and the information included in other works [20,21,22,23,24,25], refer to changes in some operating properties when a surface layer with a thickness of, at most, several hundred micrometers is affected by the processing methods by which the analyzed surfaces were made.

The results obtained by the researchers for the previously mentioned applied processing methods regarding the influence exerted on the obtained surface layer of the tribological behavior of the cylindrical surfaces, showed less information concerning the influence exerted by the burnishing and vibroburnishing processes, and these results were obtained when many actual research methods were not known. In this way, it can be considered that experimental research could highlight the evolution, over time, of the friction coefficient *µ* and lost mass Δ*m* when using certain tribological tests. Improved solutions for mathematical processing of the theoretical and experimental results could be applied.

The aim of the research, the results of which were included in the article, was to identify empirical mathematical models that highlight how distinct processing methods influence the variation, over time, in the friction coefficient and the loss of mass under the tribological conditions specified for the experimental test.

## 2. Materials and Methods

### 2.1. Theoretical Premises

It is expected that the distinct processing method affects the structure, chemical composition, and mechanical properties of the surface layer (which has a thickness of several hundred micrometers) in distinct ways.

**Figure 1:**
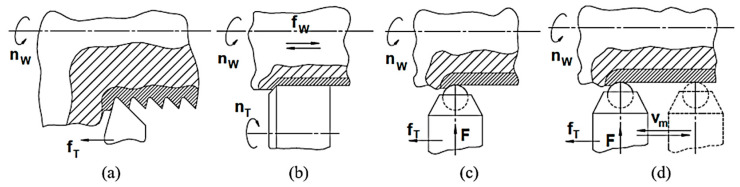
Microchanges in the surface layers after applying various processing methods: (**a**) after turning; (**b**) after grinding; (**c**) after burnishing; (**d**) after vibroburnishing.

Thus, in the case of cylindrical turning using a lathe tool, the grains from the surface layer change their shape due to the pressure exerted by the tool during the process of material removal from the workpiece. The tool used for the experiments was a SN 400 × 1000 lathe, manufactured at the Lathe Enterprise of Arad (Romania). An increase in the microhardness of the surface layer with a certain thickness can be observed, compared to the microhardness of the material that was not affected by the cutting process (Figure 1a).

The thickness of the hardened surface layer could be lower when applying a cylindrical grinding process (Figure 1b) since the forces generated by such a machining process are lower than the forces occurring during the turning process. If high cutting speeds are used in the case of turning or grinding, a possible heat-affected zone can also be generated in the surface layer of the machined part.

Considering the chemical composition of the surface layer of the test piece, the temperature reached in the processing zone, and the conditions of heat dissipation, some thermal changes could affect the microstructure of the surface layer. In this surface layer, phenomena of overheating, normalization, recrystallization, and hardening could develop, changing its mechanical properties and, as a consequence, the wear behavior of the surface layer.

In the case of the burnishing process, the burnishing tool (ball or roll) is forced to roll under a certain pressure over the entire surface that is intended to be processed, due to the relative movements performed by the workpiece and the burnishing tool, one to the other. The machining scheme presented in Figure 1c corresponds to the burnishing of a cylindrical surface when the workpiece performs a rotation movement, and the burnishing ball materializes a feed movement along the workpiece rotation axis. Generally, when applying a burnishing process, the objectives are to improve the surface roughness, increase the surface layer microhardness, and sometimes even obtain certain microrelief of the processed surface. Due to the high value of the radial force *F* exerted by the burnishing tool on the workpiece surface layer, a higher value of the hardened surface layer is expected (Figure 1c).

Compared to the common burnishing process, in the case of the vibroburnishing process, an additional vibratory movement is performed by the vibroburnishing tool, with a small amplitude and a relatively low frequency [19]. Due to the more complex movements performed by the vibroburnishing tool on the workpiece surface (Figure 1d), it is expected that certain differences concerning the microstructure and the thickness of the layer affected by the vibroburnishing process could occur, compared to the same aspects in the case of the burnishing process.

Certain microchanges develop in the surface layer due to the processing methods that involve removal of the material (e.g., the turning and grinding methods) or to the processing methods that aim to increase the microhardness and modify the surface asperities geometry. It is expected that distinct behaviors of the processed surface layers will be obtained under the conditions of tribological tests.

### 2.2. Experimental Conditions

**Figure 2:**
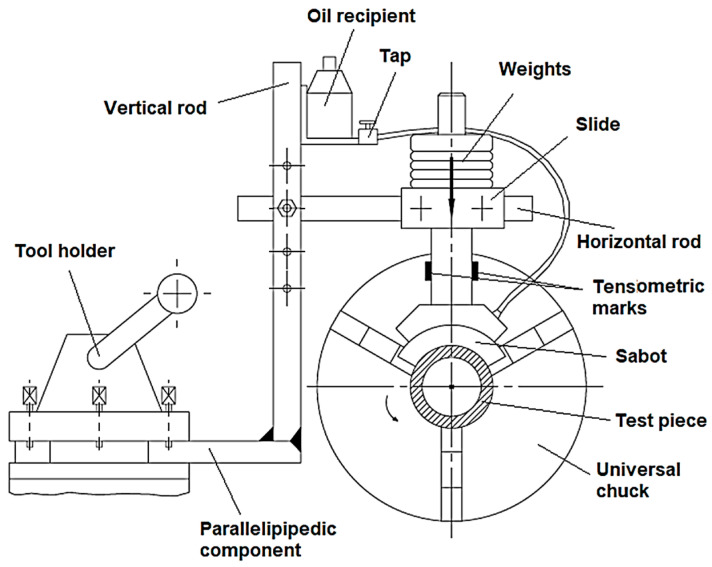
Equipment for testing the wear behavior of surfaces obtained by distinct processing methods.

To investigate the evolution of the friction coefficient value and the mass lost by the test piece during a tribological test, equipment presented in Figure 2 was used. The equipment was adapted on a universal lathe of medium size.

It can be observed that the conditions of the tribological test present certain similitudes with the conditions specific to the processes that develop in the first period of operation, when there are relative movements between the surfaces of two parts found in contact. It is known that regardless of the initial roughness, after a certain period, the values of surface roughness correspond to the local tribological conditions (local operation conditions) and these values are maintained along with the normal service period of the part.

In the case of equipment for investigating the tribological behavior of the surface layer previously obtained by different processing methods, in the lathe tool holder, a rigid frame obtained by welding was clamped. The rigid frame has a parallelepipedal component for clamping in the lathe tool holder instead of the common lathe tool. A horizontal rod could be attached, using a cylindrical joint, to the vertical component of the rigid frame. In the vertical component of the rigid frame, there are many holes where the bolt of the cylindrical joint could be placed to adapt equipment in the cases of distinct diameters of the test pieces. Along the horizontal rod, a slide could be moved and clamped in an adequate position to the vertical plane of the lathe main shaft axis. A vertical rod could move vertically in a hole that exists in the slide. At the lower end of the vertical rod, there is a part to which sabot support is assembled using screws. A proper sabot made of a material characterized by high hardness, high wear resistance, and certain roughness of the active surface is attached to the sabot support. The sabot is pressed onto the cylindrical surface of the test piece using weights of known values and placed on the vertical rod. The slide is supported by a nut screwed into the vertical rod.

A recipient containing mineral oil was attached to the vertical rod to ensure lubrication conditions for the zone between the sabot and the test piece. A tap ensures oil flow adjustment to the zone found between the sabot and test piece.

At its end, the test piece that has a bushing shape is positioned and clamped onto a mandrel clamped in the lathe universal chuck and the rotating live center.

On the vertical rod, tensometric marks are attached. These tensometric marks are connected in the electric circuit of a tensometric bridge, and they ensure the evaluation of the friction moment *M_t_*.

**Figure 3:**
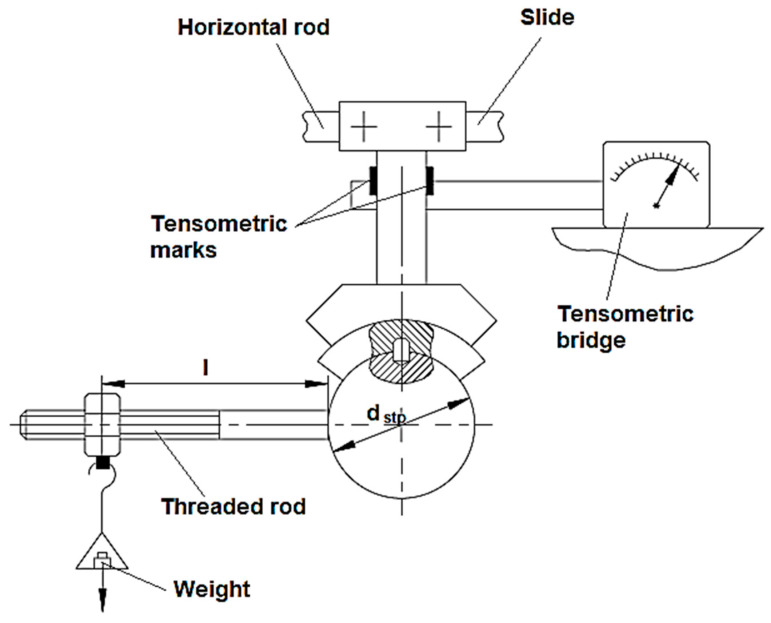
Calibration of equipment for studying the wear behavior of surfaces obtained by different processing methods.

Calibration of the equipment, including the tensometric bridge for obtaining distinct values of the friction moment *M_t_,* was performed using the working scheme from Figure 3. It can be observed that a cylindrical stud was used to solidarize the sabot with a special test piece and an initially horizontal threaded rod was attached to the special test piece; the calibration operation was then performed. Different weights of known mass values were attached to the free end of the threaded rod. Thus, they generated different torsion moments, *M_t_*. The value of the torsion moment *M_t_* applied to the test piece was measured using an adequate apparatus based on the use of a tensometric bridge.

**Figure 4:**
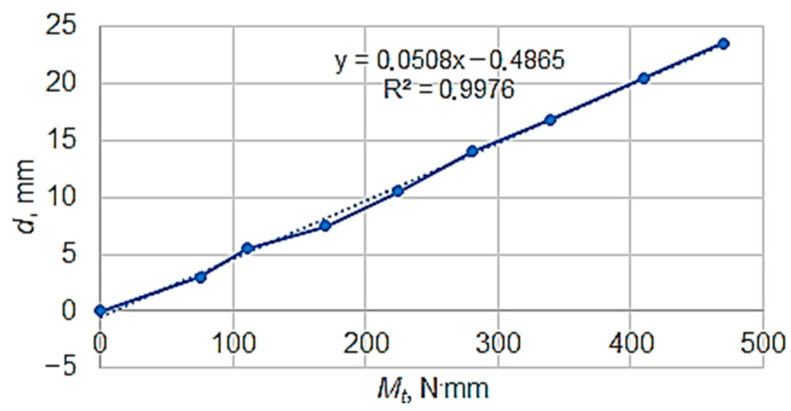
Calibration diagram for the indication *d* of the pressure measuring apparatus depending on the torsion moment *M_t_*.

The resulting calibration diagram is presented in Figure 4. In the diagram, the average values of the obtained results upon charging and discharging the equipment were taken into consideration. The diagram was made using Excel software, which made it possible to inscribe the linear function and the value of the coefficient of determination *R*^2^ corresponding to that function on the diagram.

The torsion moment generated by the mass *m* of the weight attached to the free end of the threaded rod is given by the following equation:

(1) $$M_{t}=mg\left(l+\frac{d}{2}\right)$$

where *g* is the gravitational acceleration (mm/s^2^), *m* is the mass of the weight used in each test, *l* is the length of the threaded rod (mm), and *d* is the diameter of the special test piece, expressed in mm.

Knowing the values of the torsion moment *M_t_*, the values of the friction coefficient *µ* could be calculated as follows:

(2) $$\mu =\frac{2M_{t}}{mgd}$$

During the experimental tests, weights of known mass values, *m,* were placed on the vertical rod to generate different pressures exerted by the sabot on the test piece.

The values of pressure were evaluated using the following equation:

(3) $$p=\frac{F}{A}$$

where *A* is the projection of the work surface area of the sabot in a horizontal plane.

Values of the pressure, 14,400–143,300 Pa, were obtained using weights with masses of 1–10 kg.

The test piece had an 800 rev/min rotation speed, corresponding to a peripheral speed of *v* = 75 m/min. The test pieces were made of steel 1.0060 (characterized by a tensile strength of 650–880 MPa). The test piece dimensions were as follows: the external diameter *d_ext_* = 30 mm, the internal diameter *d_int_* = 22 mm, and length *l_tp_* = 22 mm. The sabot material was X 210 Cr 12 (1.2080). The test pieces were weighted after each experimental test, which had a duration of 30 min. The total duration of a test that used a certain combination of process input factors was 210 min.

The following four processing methods were used to generate the external cylindrical surfaces, aiming to obtain information concerning the tribological behavior of surfaces generated by different machining methods:

- Turning with a rotation speed of 500 rev/min (*v* = 47.1 m/min), a longitudinal feed *f_l_* = 0.1 mm/rev and a depth of cut *a_p_* = 0.2 mm. As a result, roughness characterized by values *Ra* = 1.23–1.35 µm was generated on the turned surface;
- Grinding (using parts prepared previously by turning), which led to a surface roughness *Ra* = 0.74–0.7 µm;
- Ball burnishing applied after turning with a burnishing force *F_b_* = 400 N, rotation speed *n* = 500 rev/min (*v* = 47.1 m/min), burnishing feed *f_l_* = 0.096 mm/rev, and ball diameter *d_b_* = 15.85 mm. As a result, surface roughness characterized by *Ra* = 0.45–0.51 µm was obtained;

A so-called microrelief of 3rd category was obtained by vibroburnishing.

- Ball vibroburnishing occurs when an additional vibration characterized by the amplitude *A* = 2.6 mm and frequency *f* = 470 strokes/min (7.83 Hz) is applied to the burnishing process, as mentioned above. The other processing parameters valid in the case of the ball vibroburnishing process had the same values as those used in the case of the ball burnishing process. Microrelief of the 3rd category is characterized by a partial overlap of the grooves generated by the ball on the vibroburnished test piece surface.

**Figure 5:**
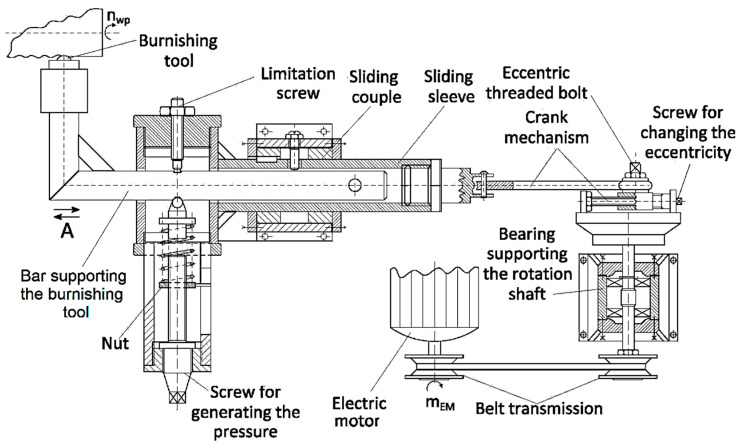
Device adapted on universal lathes to materialize vibroburnishing and oscillatory burnishing of the workpiece surface layer [26].

For ball burnishing and ball vibroburnishing, equipment schematically presented in Figure 5 was used. The processing scheme involved the rotation of the test piece, while the ball used as a vibroburnishing tool performed a vibration movement in a parallel direction to the test piece axis. This vibration movement was obtained by using an electric motor whose rotation movement was transformed into a rectilinear alternative movement of the ball support using a mechanism-type rod-connecting rod. The pressure necessary in the vibroburnishing process was ensured by using a spring whose compression could be set by means of a nut that moves along a threaded screw.

Belt wheels with different external diameters were used to change the vibratory movement frequency. The amplitude of the vibratory movement was set, acting on the length of the rod included in the rod-connecting rod mechanism.

Seven values of the pressure exerted on the sabot, found to be between 14,400 Pa and 143,300 Pa, were considered to obtain a larger image concerning the influence exerted by the process input factors on the values of the friction coefficient *µ* and lost mass Δ*m* in the cases of surfaces generated by previously applying four different machining processes. The range of surface pressures for experimental research was established based on some information identified in the consulted literature. The results of preliminary experimental research and the possibilities of the equipment on which the experimental research was carried out were also considered, with the aim of obtaining sufficiently differentiated values of the output parameters, to increase the confidence level of the proposed mathematical models.

Appreciating that the test duration *t* could significantly affect the values of the process output parameters, the values of the friction coefficient *µ* and lost mass Δ*m* were measured every 30 min for the total test duration, which was 210 min. When investigating the influence of the test duration *t* on the variation in the friction coefficient *µ* in the preliminary experimental tests, it was found that the values of the friction coefficient *µ* stabilized after about 90 min. However, to increase the confidence level of the experimental results, longer test durations were used, i.e., 210 min.

Two experimental tests were established for each experiment characterized by the imposed values of the sets of process input factors. Only the average value obtained for the process output parameters in the two experiments are included in Table 1.

### 2.3. Modeling the Tribological Test Using the Finite Element Method

Starting from the schematic representation of the equipment used to test the wear behavior in Figure 2, a simulation based on ANSYS software for finite element analysis was considered. The aim was to highlight some aspects that characterize the wear test process. The materials of the two components directly participating in the wear process were those used in the experimental research (steel 1.0060 as the test piece and steel X210Cr12 for the sabot). The two surfaces in contact were considered under ideal conditions, so the heights of the roughness in the process were not considered.

We started by evaluating the amount of material removed from the test piece under the pressure *p* = 14,400 Pa and coefficient of friction *µ* = 0.127. The sabot was considered to be pushed down by a force of 162.86 N, which corresponds to a uniform pressure of 14,400 Pa. The results obtained correspond to a transient analysis for a full revolution time stepped in 18 equal intervals.

The amount of material removed from the test piece was determined by taking into account the volume of the test piece before the start of the process and the volume of the test piece after one rotation. In the simulation process, APDL-type POST commands were used for this purpose.

Limitations in computing power forced us to set just a 1 mm element size for meshing purposes. Considering a tensile yield strength of 310 MPa in the case of the test piece material, equivalent (von Mises) stresses were set to all parts.

**Figure 6:**
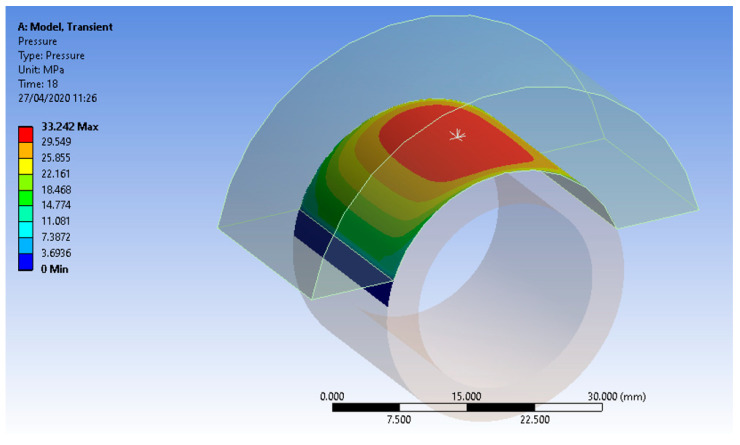
The pressure exerted by the sabot on the test piece surface.

**Figure 7:**
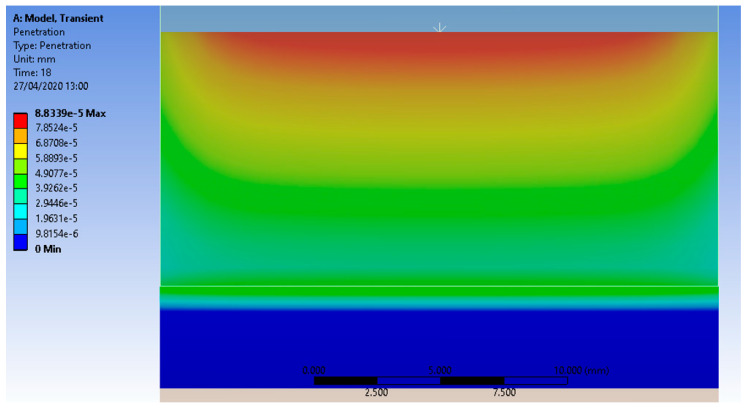
Lowering the sabot (penetration) as a consequence of the wear process that affects the test piece.

**Figure 8:**
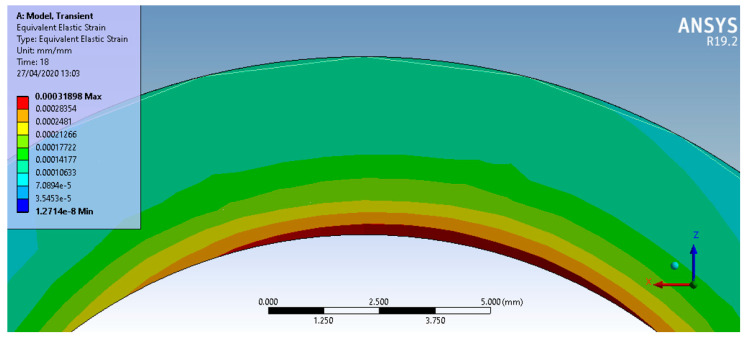
Equivalent stresses (von Mises) developed in the test piece.

As a result of applying the finite element analysis method, the graphical representations from Figure 6, Figure 7 and Figure 8 were developed. Thus, in Figure 6, the variation in the pressure on the contact surface between the sabot and the test piece with a bushing shape was highlighted. Higher pressure values can be observed near the axis of the rod through which the sabot is pressed on the outer cylindrical surface of the test piece. The contact pressure peaked at just 10% of the yielding value.

Figure 7 took into account a survey of the penetration of the sabot into the test piece material due to the evolution of the wear process. The APDL POST commands have given a new microvolume value for the test piece, which is about 0.003 mm^3^ smaller than the initial microvolume.

In Figure 8, the variation in the equivalent stresses (von Mises) can be observed, appreciating that they could affect, possibly even to a small extent, the removal of material from the test piece due to the wear process. The yielding value was not reached for the steel 1.0060 ST 60-2. This essentially means that the elastic state of the material was not exceeded, and there was only a small amount of material flow evaluated as being below 0.1%.

## 3. Results

The experimental results included in Table 1 were mathematically processed using specialized software, based on the method of least squares [27]. The software facilitates the selection of the most convenient empirical mathematical model using Gaussian criterion. This criterion considers the value of the so-called Gauss sum, which is determined as the sum of the squares of the differences between the ordinates of the points that correspond to the selected empirical mathematical model, and those determined experimentally for the same values of the abscissas. The lower the value of Gauss’s criterion is, the more convenient the considered empirical mathematical model is to the experimental results.

As mentioned above, the software ensures the selection of an adequate empirical mathematical model, taking into consideration five such models (polynomial of the first and second degree, power-type function, exponential-type function, and hyperbolic function). For all eight groups of experimental results (that correspond to the sizes of the friction coefficient *µ* and the lost mass Δ*m* in the cases of the four previously applied processing methods), the software showed that the most appropriate empirical mathematical model is a polynomial of the second degree (for which the minimum value of the Gauss’s criterion was determined).

For example, in the case of turning, such an empirical mathematical model is as follows:

(4) $$\mu =0.209+2.766\times 10^{-7}p-1.304\times 10{\;}^{-13}p^{2}+8.117\times 10^{-5}t+7.970\times 10^{-9}t^{2}$$

for which the Gauss’s coefficient has the value *S_G_* = 4.336001 × 10^−6^.

On the other hand, it can be noticed that the empirical mathematical model of a polynomial of the second degree does not allow an immediate image, concerning the influence exerted by each of the process input factors on the values of the friction coefficient *µ* and lost mass Δ*m*, to be formed. For this reason, the power-type functions were preferred, since, in the case of such functions, the values of the exponents attached to the sizes corresponding to the process input factors offer immediate information regarding the intensity of the influence exerted by each of the considered process input factors on the values of the output parameters.

In this way, the following empirical mathematical model was determined for the friction coefficient *µ* corresponding to the tested turned surface:

(5) $$\mu =0.123p^{0.0593}t^{0.00335}$$

for which the value of the Gauss’s criterion is *S_G_* = 3.825921 × 10^−5^.

It can be observed that the value of Gauss’s criterion determined when using the power-type function is higher than the value of the same criterion when using a polynomial-type function of the second degree. Still, we considered that the advantages of the immediate obtainment of an image concerning the influence exerted by the process input factors on the parameter of technical interest (in this case, the friction coefficient *µ*) are more important. Subsequently, only the power-type functions were used.

By mathematical processing of the experimental results included in Table 1, the following empirical mathematical models have been determined thus far:

- In the case of surfaces obtained by turning, the following model was determined:

(6) $$∆m=4.285p^{0.0444}t^{0.442}$$

(*S_G_* = 5.982294);

- In the case of the surfaces obtained by grinding, the following models were determined:

(7) $$\mu =0.121p^{0.0554}t^{0.00387}$$

(*S_G_* = 4.357229 × 10^−5^);

(8) $$∆m=3.632p^{0.0335}t^{0.486}$$

(*S_G_* = 8.259409);

- In the case of surfaces obtained by burnishing, the following models were determined:

(9) $$\mu =0.107p^{0.0471}t^{0.00285}$$

(*S_G_* = 7.776968 × 10^−6^);

(10) $$∆m=3.308p^{0.0348}t^{0.459}$$

(*S_G_* = 11.31938);

- In the case of vibroburnished surfaces, the following models were determined:

(11) $$\mu =0.0634p^{0.0708}t^{0.00295}$$

(*S_G_* = 1.884871 × 10^−4^);

(12) $$∆m=0.876p^{0.0292}t^{0.672}$$

(*S_G_* = 15.76152).

**Figure 9:**
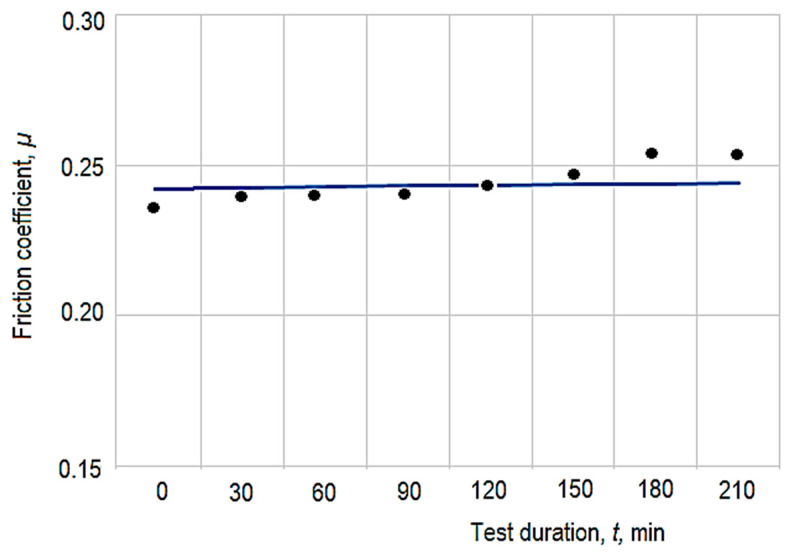
Testing the validity of the empirical mathematical model defined by Equation (6) (*p* = 76,600 Pa).

The empirical mathematical model defined by Equation (6) was determined by taking into account the experimental values of the friction coefficient *µ* recorded in columns 4, 5, 6, 8, 9, and 10 in Table 1 (except, therefore, when determining the model mathematically; the results are in column 7 in this case). The graphical representation from Figure 9 was developed to verify the validity of the empirical mathematical model defined by Equation (6). In this graphical representation, the continuous curved line corresponds to the mathematical model defined by Equation (6). The points in the diagram in Figure 6 correspond to the values of the experimental results recorded in column 10 in Table 1. It can be observed that the values of the coefficient of friction µ are near to the curved line, both above and below it. This confirms the validity of the empirical model determined by mathematical processing of the experimental results, since the points in the diagram represent results that were not used in determining the empirical mathematical model.

**Figure 10:**
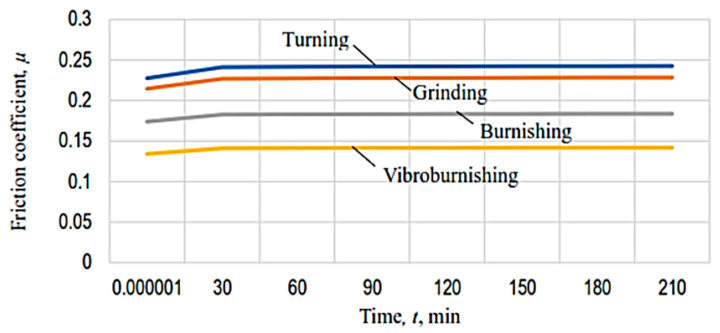
The influence exerted by the process duration on the value of the friction coefficient µ for different machining processes applied to the tested surface (*p* = 70,000 Pa).

**Figure 11:**
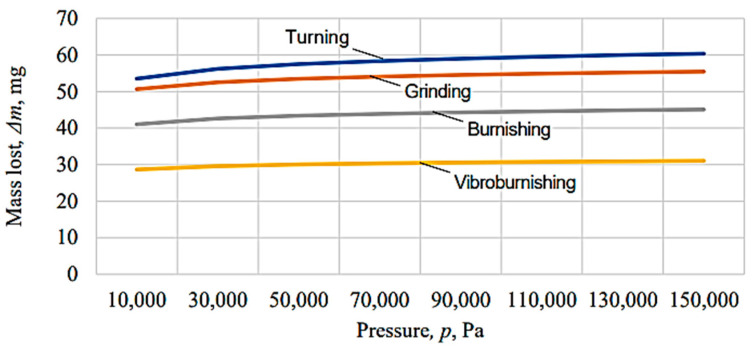
Influence exerted by the pressure on the mass lost Δ*m* for different machining processes applied to the tested surface (*t* = 150 min).

**Figure 12:**
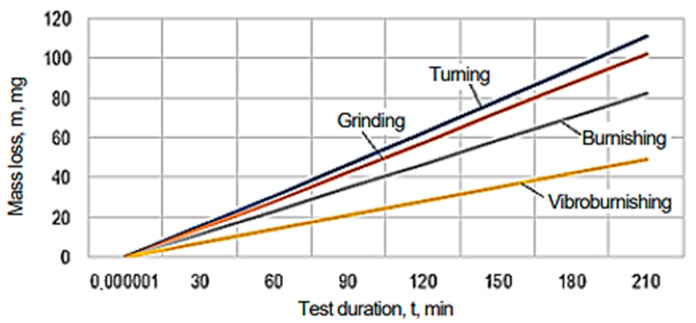
The influence exerted by the process duration *t* on the mass lost Δ*m* for different machining processes applied to the tested surfaces (*p* = 70,000 Pa).

**Figure 13:**
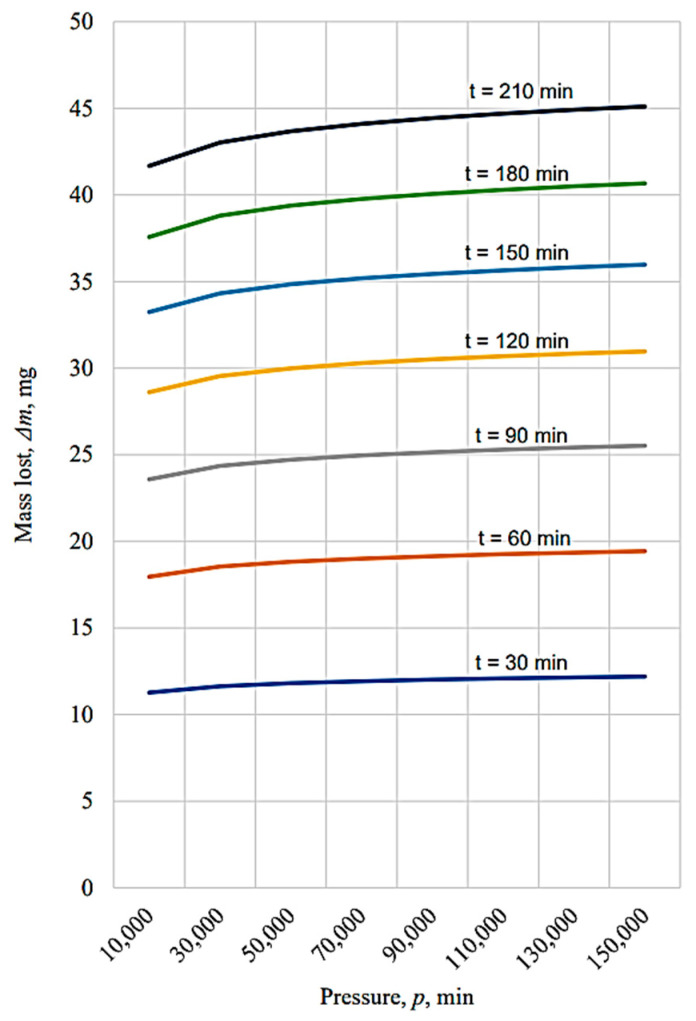
The influence exerted by the pressure on the mass lost Δ*m* for different durations of the process applied to the tested surface in the case of the vibroburnishing process.

**Figure 14:**
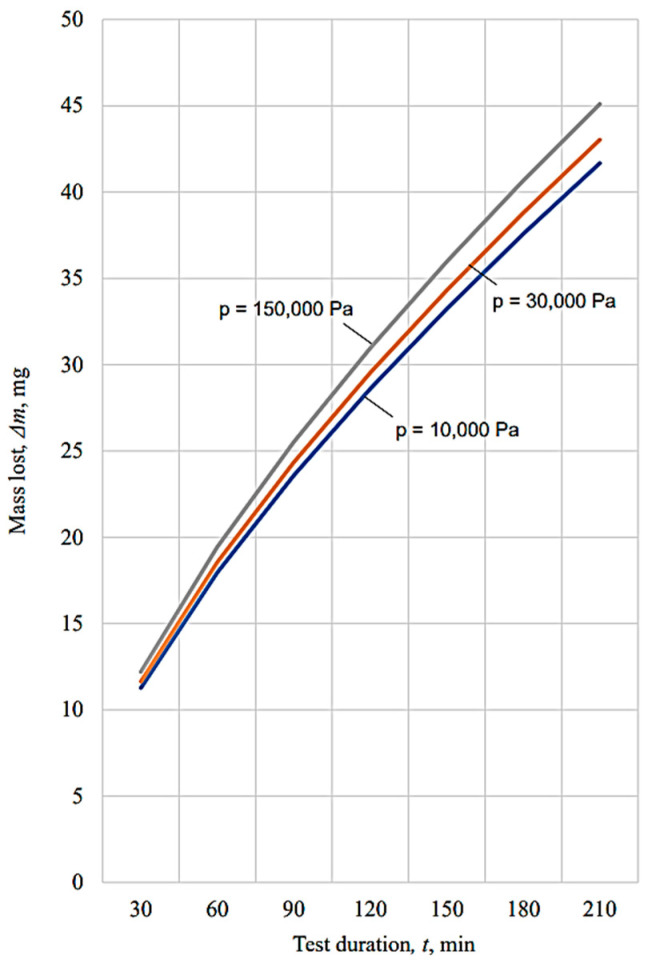
Influence exerted by the process duration *t* on the mass lost Δ*m* for different pressures *p* applied to the tested surface in the case of the vibroburnishing process.

**Figure 15:**
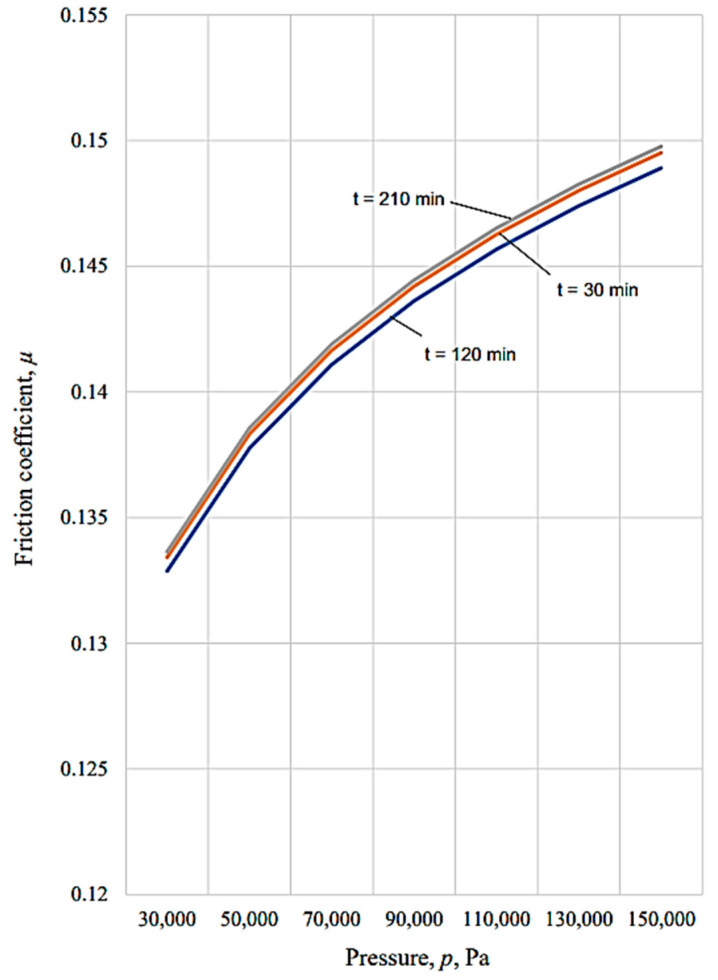
Influence exerted by the pressure *p* on the friction coefficient µ for different process durations *t* in the case of vibroburnishing applied to the tested surface.

**Figure 16:**
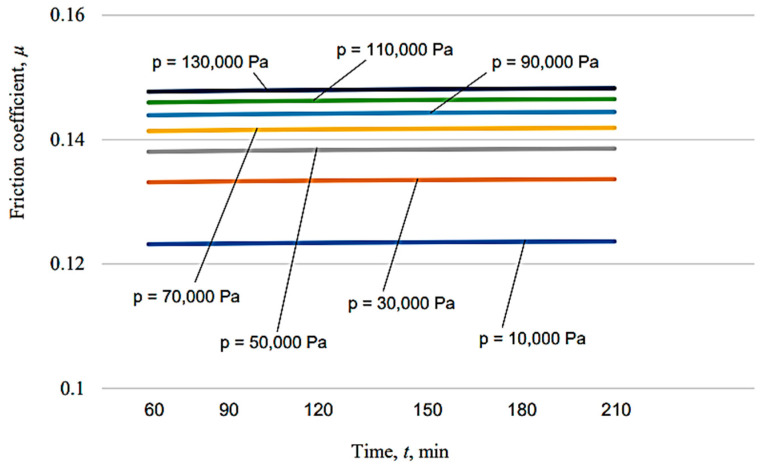
Influence exerted by the process duration *t* on the friction coefficient *µ* for different pressures *p* in the case of vibroburnishing applied to the tested surface.

The graphical representations in Figure 10, Figure 11, Figure 12, Figure 13, Figure 14, Figure 15 and Figure 16 were achieved by considering the empirical mathematical models defined by Equations (5)–(12).

## 4. Discussion

Some remarks could be formulated by examining the empirical mathematical models from Equations (5–12) and the graphical representations in Figure 10, Figure 11, Figure 12, Figure 13, Figure 14, Figure 15 and Figure 16.

Thus, the similitude of the empirical mathematical models to the empirical mathematical models that represent the variation in the friction coefficient *µ* and lost mass Δ*m* could be observed for all the test pieces obtained by different processing methods. In all eight empirical mathematical models, the low values of the exponents attached to the pressure *p* show that this process input factor (pressure, *p*) exerts a certain influence on the friction coefficient *µ* and lost mass Δ*m*, but this influence is low enough. As expected, the increase in pressure, *p,* is accompanied by an increase in the friction coefficient *µ*, due to the increase in the normal force *F* exerted on the contact surface between the test piece and sabot.

On the other hand, the influence exerted by the test duration *t* on the friction coefficient *µ* is also low, and is even less than the influence exerted by the pressure *p*, since the values of the exponents attached to the duration test *t* in the empirical mathematical models defined by Equations (5)–(12) are lower than the values of the exponents attached to the pressure *p* in the same mathematical equations.

In contrast to the influence exerted on the friction coefficient *µ*, in the case of the lost mass Δ*m*, it can be noticed that the duration test *t* exerts a stronger influence, since the values of the exponents attached to the test duration *t* are higher than the values of the exponents attached to the pressure *p* in the same mathematical equations. As expected, the increase in the test duration *t* causes an increase in the lost mass Δ*m*.

The diagrams presented in Figure 10, Figure 11, Figure 12, Figure 13, Figure 14, Figure 15 and Figure 16 show that the decrease in the initial values of the surface roughness parameter *Ra,* which corresponds to the four surfaces obtained using different processing methods, leads to a decrease in the values of the friction coefficient *µ* and lost mass Δ*m*. In the case of the friction coefficient *µ*, this aspect is justified as it is known that, generally, the value of the coefficient *µ* diminishes when the heights of the surface micro asperities are lower.

Similar arguments could also be valid in the case of the change in the value of the lost mass Δ*m* when the height of the surface asperities is lower. Indeed, when the heights of the surface micro asperities are higher, the local pressure on the micro asperities found in contact is higher, and a higher quantity of the test piece materials could be removed.

## 5. The Correlation between the Values of the Friction Coefficient and Lost Mass When Applying Tribological Tests

The inverse evolution of the two process output parameters analyzed in this paper showed a correlation between them. To find the answer to this question, the correlation coefficient value was determined, considering that the values of both output parameters (friction coefficient *µ* and lost mass Δ*m*) were established within the same sets of experiments.

It is known that the value of the coefficient of the correlation (the Pearson’s correlation coefficient *r_xy_* for the uncorrected, non-standard form) is given by the following [28]:

(13) $$r_{xy}=\frac{n\sum x_{i}y_{i}-{\sum x}_{i}\sum y_{i}}{\sqrt{n\sum x_{i}{}^{2}-\left(\sum x_{i}\right){}^{2}}\sqrt{n\sum y_{i}{}^{2}-{\left(\sum y_{i}\right)}^{2}}}$$

where *n* corresponds to the number of measurements found in each of the two comparison sets of the measured values *x_i_* and *y_i_* when *i* = 1, 2, …, *n*. It is usual to consider a strong correlation between the two series of considered values if the Pearson’s coefficient is close to 1.00 or −1.00, and a low correlation when the Pearson’s coefficient has a value close to zero.

The function CORRELATION from the Excel software was used to evaluate the correlation between the values of the friction coefficient *µ* and the lost mass Δ*m* in the cases of the surfaces previously obtained by applying different processing methods. The determined Pearson’s coefficient values when applying the four different processing methods applied previously to the tested surface were the following:

*r_xy_* = 0.534322 for turning;
*r_xy_* = 0.617232 for grinding;
*r_xy_* = 0.531417 for ball burnishing;
*r_xy_* = 0.34025 for ball vibroburnishing.

Following the existing conventions concerning the evaluation of the correlation using the Pearson’s coefficient, this means that, in the cases of all the different processes applied to the test piece surfaces before testing their tribological behavior, there is an average correlation between the compared sizes in the case of the ball vibroburnishing process, and a strong correlation in the cases of turning, grinding, and ball burnishing. The maximum value of Pearson’s coefficient corresponds to the grinded surface, while the minimum value of Pearson’s coefficient was found for the ball vibroburnished surface.

## 6. Conclusions

The tribological characteristics of the surfaces can be influenced, to a significant extent, by the processing technique by which the respective surfaces were obtained. Testing equipment, adapted on a universal lathe, was designed and materialized to mathematically model the variation in the friction coefficient and the lost mass. The equipment involves pressing a sabot made of hard material onto the external cylindrical surface of a rotating test piece made of the material to be tested. The experimental research was carried out on steel 1.0060 test pieces; the mechanical treatment by burnishing and vibroburnishing could improve the tribological characteristics more compared with the surfaces obtained by turning and grinding. Mathematical models using the power-type function have been determined to highlight the influence of pressure and test duration on the size of the friction coefficient and the microamount of material removed from the test piece due to the tribological test. It was considered that the change in the tribological behavior of the test piece material is a result of the change in the microstructure of the material in the surface layer; the shape and dimensions of the micro asperities result from the previous application of certain processing methods. Information was obtained regarding the intensity of the variation in the friction coefficient and mass loss depending on the applied pressure and the experiment test duration. It can be noticed that, essentially, the influence exerted by the pressure and test duration is relatively low in the case of the friction coefficient. If the mass loss is analyzed, it can be noticed that the test duration has a stronger influence than the influence exerted by the pressure for the considered experimental conditions. Among the investigated processing techniques, burnishing and vibroburnishing had the most convenient results for the friction coefficient and mass microamount removed from the test piece.

## Figures and Tables

**Table 1:** Experimental conditions and results.

Line no.1	Applied Processing Method	Testing Pressure, *p*, Pa	Friction Coefficient, *µ*, for a Duration Test, *t* (min):								Mass Lost by the Test Piece, Δ*m* (mg), for a Test Duration *t* (min)							
2			0	30	60	90	120	150	180	210	0	30	60	90	120	150	180	210
Column no.1	2	3	4	5	6	7	8	9	10	11	12	13	14	15	16	17	18	19
4	Turning	14,400	0.212	0.217	0.220	0.223	0.226	0.229	0.233	0.234	0	27.5	41.2	49.0	57.8	62.8	66.2	72.0
5		33,120	0.219	0.222	0.225	0.227	0.230	0.233	0.237	0.238	0	28.3	43.3	51.2	58.5	63.4	67.3	72.5
6		59,300	0.225	0.229	0.230	0.233	0.236	0.238	0.243	0.243	0	29.1	44.2	52.0	59.5	64.0	68.4	73.0
7		76,600	0.233	0.238	0.241	0.242	0.243	0.249	0.255	0.255	0	30.0	45.0	53.0	60	64.5	70.1	73.5
8		103,800	0.237	0.243	0.244	0.245	0.247	0.253	0.260	0.261	0	31.2	45.8	53.8	61.0	64.9	70.9	73.9
9		123,900	0.241	0.247	0.248	0.250	0.251	0.255	0.263	0.264	0	33.0	46.7	55.0	61.8	65.4	71.5	74.3
10		143,300	0.244	0.252	0.253	0.254	0.255	0.258	0.265	0.266	0	33.8	47.9	55.9	62.4	66.0	71.9	74.7
11	Grinding	14,400	0.198	0.203	0.207	0.210	0.214	0.217	0.220	0.221	0	24.1	38.2	47.0	55.1	59.5	63.7	66.1
12		33,120	0.205	0.207	0.211	0.216	0.218	0.222	0.226	0.227	0	24.7	39.3	47.8	55.8	59.9	64.6	66.5
13		59,300	0.211	0.213	0.215	0.223	0.226	0.229	0.233	0.234	0	25.4	39.7	49.1	56.3	60.5	65.0	66.9
14		76,600	0.216	0.218	0.219	0.227	0.230	0.232	0.239	0.241	0	26.0	40.0	50.0	57.0	61.0	65.6	67.2
15		103,800	0.220	0.225	0.224	0.230	0.234	0.238	0.244	0.245	0	26.7	40.6	50.7	57.8	61.4	66.2	67.5
16		123,900	0.226	0.229	0.231	0.233	0.237	0.241	0.245	0.248	0	27.3	41.5	51.2	58.2	61.7	66.9	67.8
17		143,300	0.229	0.233	0.236	0.239	0.242	0.245	0.248	0.250	0	28.2	42.4	51.9	59.2	62.0	67.5	68
18	Burnishing	14,400	0.164	0.167	0.170	0.172	0.173	0.174	0.175	0.175	0	19.0	32.1	41.0	45.7	48.1	50.1	51.0
19		33,120	0.168	0.171	0.174	0.176	0.177	0.178	0.178	0.179	0	19.5	32.9	41.7	46.4	48.3	50.5	51.3
20		59,300	0.172	0.175	0.178	0.180	0.181	0.181	0.182	0.183	0	20.2	33.9	42.5	47.0	48.6	50.9	51.6
21		76,600	0.175	0.178	0.181	0.183	0.185	0.185	0.186	0.186	0	21.0	35.0	43.0	47.5	49.0	51.3	51.9
22		103,800	0.178	0.181	0.184	0.186	0.187	0.188	0.190	0.191	0	21.3	35.6	43.4	47.8	49.3	51.6	52.1
23		123,900	0.181	0.184	0.187	0.189	0.191	0.192	0.192	0.193	0	21.9	35.9	43.8	48.0	49.5	51.9	52.3
24		143,300	0.184	0.187	0.190	0.192	0.193	0.194	0.194	0.195	0	23.1	37.8	44.0	48.2	49.7	52.2	52.4
25	Vibroburni-shing	14,400	0.124	0.127	0.129	0.130	0.130	0.131	0.131	0.131	0	10.0	17.6	27.0	35.9	36.6	37.0	37.5
26		33,120	0.127	0.130	0.132	0.133	0.133	0.133	0.133	0.133	0	10.3	18.2	27.3	36.3	36.8	37.3	37.7
27		59,300	0.131	0.134	0.136	0.137	0.137	0.137	0.137	0.137	0	10.6	18.8	27.5	36.6	37.0	37.6	37.9
28		76,600	0.135	0.136	0.138	0.139	0.139	0.139	0.139	0.139	0	11.0	19.0	28.0	36.9	37.2	37.8	38.0
29		103,800	0.138	0.141	0.143	0.144	0.144	0.144	0.144	0.144	0	11.3	19.3	28.3	37.2	37.3	37.9	38.1
30		123,900	0.140	0.143	0.147	0.148	0.148	0.149	0.149	0.149	0	11.4	19.4	28.6	37.3	37.5	38.1	38.2
31		143,300	0.141	0.144	0.148	0.149	0.149	0.150	0.150	0.150	0	11.9	19.6	28.9	37.4	37.6	38.2	38.3

## Data Availability

Not applicable.
